# The relationship between belief in a just world and prosocial behavior: the role of psychological resilience and empathic capacity

**DOI:** 10.3389/fpsyg.2025.1520451

**Published:** 2025-03-14

**Authors:** Cong Liu, Wenshu Fan, Qiuyu Tan, Kai Yun, Wei Huang

**Affiliations:** ^1^Sichuan Zhang Daqian Research Center, Neijiang Normal University, Neijiang, China; ^2^Mental Health Education Center for College Students, Chengdu University of Traditional Chinese Medicine, Chengdu, China; ^3^Department of Basic Medicine, Chengdu University of Traditional Chinese Medicine, Chengdu, China; ^4^School of Economics, Southwest Minzu University, Chengdu, China; ^5^College of Preschool and Primary Education, China West Normal University, Nanchong, China

**Keywords:** prosocial behavior tendency, empathic capacity, belief in a just world, psychological resilience, moderated mediation

## Abstract

**Objective:**

This study investigated how belief in a just world (BJW) influences prosocial behavior tendency (PBT) through psychological resilience (PR), and examined how empathic capacity (EC) moderates this mediation process.

**Method:**

Data were collected from 955 Chinese university students (Mage = 19.5 years, SD = 1.3; 65.6% female) using validated scales measuring BJW, PBT, PR, and EC.

**Results:**

Three key findings emerged: (1) BJW positively predicted PBT both directly (*β* = 0.301, *p* < 0.001) and indirectly through PR; (2) EC moderated the relationship between BJW and PR, with the positive association being stronger for individuals with lower EC (*b* = 0.45, *p* < 0.001) compared to those with higher EC (*b* = 0.23, *p* < 0.01); (3) The indirect effect of BJW on PBT through PR was stronger for individuals with lower EC, indicating a moderated mediation effect.

**Conclusion:**

These findings advance our understanding of prosocial behavior by identifying distinct pathways through which beliefs and emotional capacities interact. The results suggest that interventions to promote prosocial behavior should be tailored based on individual differences in empathic capacity, with different approaches needed for high versus low EC individuals.

## 1 Introduction

Prosocial behavior has long been a subject of interest for researchers in psychology, sociology, and related fields. It encompasses a wide range of actions, from everyday acts of kindness to more substantial forms of helping behavior, such as volunteering and charitable giving (Lim and DeSteno, 2016). The importance of understanding the factors that influence prosocial behavior has been highlighted by its numerous benefits, both for individuals and society as a whole. For example, engaging in prosocial behavior has been linked to increased well-being, better mental health, and stronger social connections (Curry et al., 2018; Hui et al., 2020). Moreover, prosocial behavior is essential for building and maintaining cohesive communities and promoting social harmony (Biglan et al., 2020). This is particularly relevant in Chinese society, where rapid social transformation and economic development have led to changing patterns of social interaction and support among young adults, especially university students (Zhang et al., 2024).

Among the various psychological factors that have been examined in relation to prosocial behavior, belief in a just world (BJW) has emerged as a notable construct. BJW refers to the belief that the world is a fair and orderly place where people generally get what they deserve (Lerner, 1980). Individuals with a strong BJW tend to believe that good deeds are rewarded and bad deeds are punished, which can influence their attitudes and behaviors toward others (Hafer and Sutton, 2016). This belief system may be particularly salient in Chinese culture, where traditional values emphasizing karma and moral reciprocity align closely with just-world beliefs (Tian et al., 2019). Several studies have investigated the relationship between BJW and prosocial behavior, with mixed results. While some research has found a positive association between BJW and prosocial behavior (Bègue et al., 2008; Jiang et al., 2017), others have reported no significant relationship or even a negative association (Ucar et al., 2019). These inconsistent findings suggest that the relationship between BJW and prosocial behavior may be more complex than initially thought and may be influenced by other factors, such as psychological resilience and empathic capacity.

The context of Chinese university students provides a unique and important setting for examining these relationships. First, Chinese university students face distinct pressures and challenges, including intense academic competition, career uncertainty, and the need to balance traditional values with modern aspirations (An et al., 2024). Second, they represent a generation that has grown up during China’s rapid social and economic transformation, potentially influencing their beliefs about justice and their approach to prosocial behavior (Chobthamkit et al., 2024). Despite the growing body of research on the relationship between BJW and prosocial behavior, there are still significant gaps in our understanding of this topic within this specific context. First, the role of psychological resilience as a potential mediator in the relationship between BJW and prosocial behavior has not been thoroughly investigated. Psychological resilience, which refers to an individual’s ability to adapt and cope with adversity and stress (Fletcher and Sarkar, 2013), has been shown to be positively associated with prosocial behavior (Datu and Restubog, 2020; Kındap-Tepe and Aktaş, 2021). This is particularly relevant for Chinese university students who must navigate significant academic and social pressures while maintaining their psychological well-being. Second, the moderating role of empathic capacity in the relationship between BJW and prosocial behavior has not been adequately explored. Empathic capacity, which involves the ability to understand and share the emotions of others, has been consistently linked to prosocial behavior (Decety et al., 2016; Van der Graaff et al., 2018). Understanding these relationships in the Chinese university context is crucial for developing culturally appropriate interventions to promote prosocial behavior and psychological well-being among this population.

### 1.1 The direct effect of belief in a just world on prosocial behavior tendency

The relationship between Belief in a Just World (BJW) and Prosocial Behavior Tendency (PBT) can be understood through Conservation of Resources (COR) theory (Hobfoll, 1989), which positions BJW as a key cognitive resource that facilitates prosocial engagement. Within this resource-based framework, BJW functions as a fundamental psychological resource that individuals can leverage to maintain and enhance their capacity for prosocial behavior (An et al., 2024; Guo et al., 2022; Reinhardt et al., 2023).

COR theory suggests that individuals strive to obtain, retain, and protect resources that enable goal achievement and well-being (Hobfoll, 1989). BJW serves as such a resource by providing cognitive frameworks that support prosocial action through multiple mechanisms. First, as demonstrated by just-world theory (Lerner, 1980), BJW acts as a resource that helps individuals maintain cognitive consistency when faced with others’ needs. When encountering situations of injustice or suffering, individuals with strong BJW experience cognitive dissonance (Festinger, 1957), motivating them to engage in prosocial behaviors to restore their sense of justice and reinforce their belief system.

Self-perception theory (Bem, 1972) complements this resource-based understanding by explaining how BJW as a resource gets reinforced through behavioral feedback. When individuals with high BJW engage in prosocial actions, they interpret these behaviors as confirmatory evidence of their beliefs, creating what Kleinke and Meyer (1990) describe as a resource enhancement cycle. This cyclical process helps explain the consistent positive relationship between BJW and PBT observed in empirical research.

Recent studies have expanded our understanding of how BJW functions as a psychological resource across various contexts. Research has examined BJW’s role in employee voice behavior (Li et al., 2014), resilience development (Lin et al., 2022), and responses to global crises like the COVID-19 pandemic (Wu et al., 2018; Li et al., 2022; Serrano-Montilla et al., 2021). These studies demonstrate how BJW serves as a stable resource that supports prosocial engagement across different situations and challenges.

The resource function of BJW has been further illuminated through moral identity and social identity frameworks. Aquino and Reed II. (2002) showed how moral identity enhances the resource potential of BJW by linking it to individuals’ self-concept. Recent research by Rullo et al. (2022) found that moral identity symbolization moderates how effectively individuals can deploy BJW as a resource for prosocial behavior, suggesting that the resource value of BJW varies with individual characteristics.

While previous research has established BJW’s role as a psychological resource, several theoretical gaps remain. First, most studies have focused on direct relationships rather than examining the resource mechanisms through which BJW enables prosocial behavior. Second, although both cognitive beliefs and emotional capacities have been identified as distinct resources, their interaction in promoting prosocial behavior remains underexplored. Third, existing research has predominantly examined these resource dynamics in Western contexts, raising questions about their cultural universality. The present study addresses these gaps by examining how BJW as a resource operates through psychological resilience, interacts with empathic capacity, and functions within Chinese cultural contexts.

Based on COR theory and the substantial evidence demonstrating BJW’s role as a prosocial resource, we propose: Hypothesis 1: An individual’s Belief in a Just World (BJW) positively relates to their Prosocial Behavior Tendency (PBT).

### 1.2 The mediating role of psychological resilience

Within the Conservation of Resources (COR) framework, psychological resilience (PR) functions as a dynamic resource-building mechanism that transforms initial resources like BJW into sustained capacities for prosocial action. COR theory posits that individuals not only strive to protect existing resources but also invest them to develop new resource reservoirs (Hobfoll, 1989). In this process, BJW serves as a primary resource that enables the development of psychological resilience, which in turn facilitates prosocial behavior through enhanced resource management capabilities.

The resource development process from BJW to psychological resilience operates through multiple mechanisms identified by COR theory. First, individuals with strong BJW possess cognitive resources that allow them to view challenges as temporary and manageable rather than as permanent threats to their resource base (Lin et al., 2022). This cognitive appraisal pattern facilitates resilience development by promoting adaptive resource conservation strategies. Second, BJW creates a sense of predictability and control that serves as a foundational resource for building psychological resilience (Wu et al., 2018).

Research consistently demonstrates how BJW functions as a resource catalyst for psychological resilience across various contexts. Studies have found that individuals with higher BJW demonstrate enhanced resource development through greater adversity adaptation, more effective stress management resource deployment (Li et al., 2022), and improved emotional regulation resource utilization (Zhang et al., 2020). These findings align with COR theory’s principle that initial resources facilitate the acquisition of additional resources.

The connection between psychological resilience and prosocial behavior can be understood through COR theory’s resource investment principle. When individuals possess greater psychological resilience, they have more resources available to invest in prosocial actions. PR functions as what Hobfoll (2011) terms a “resource caravan,” providing individuals with multiple resources they can deploy in helping situations. Resilient individuals are better equipped to handle the potential resource costs associated with prosocial behavior, as they maintain robust resource reserves that can be activated when needed.

Empirical evidence supports this resource-based understanding of the PR-prosocial behavior relationship. Studies have shown that resilient individuals more effectively deploy their resources across various prosocial contexts, including volunteering (Wu et al., 2018), helping behaviors (Li et al., 2022), and charitable giving (Zhang et al., 2020). This relationship appears particularly strong in challenging contexts, where resilience resources become crucial for maintaining prosocial engagement.

Based on COR theory and the empirical evidence supporting these resource dynamics, we propose: Hypothesis 2: Psychological resilience mediates the relationship between belief in a just world and prosocial behavior tendency, functioning as a resource-building mechanism.

### 1.3 The moderating role of empathic capacity

Within COR theory, empathic capacity (EC) operates as a resource multiplier that enhances the efficiency of resource conversion processes. Specifically, EC influences how effectively individuals can transform their BJW resources into psychological resilience (Hobfoll, 2002). This conceptualization of EC as a resource multiplier provides a novel theoretical explanation for its moderating role in the BJW-PR relationship within the broader resource dynamics of prosocial behavior.

EC functions as a resource multiplier through several mechanisms identified in COR theory. When individuals possess higher EC, they more effectively utilize their existing resources, particularly in social contexts (Davis, 1983). This enhanced resource utilization occurs because EC encompasses both cognitive and affective components, including perspective-taking and empathic concern (Decety and Jackson, 2004), which facilitate more efficient social resource development processes. These components enable individuals to build stronger support networks that serve as additional resource reservoirs strengthening the relationship between BJW and resilience.

Research demonstrates that individuals with higher empathic capacity show more efficient resource conversion patterns in translating their just-world beliefs into psychological adjustment and well-being (Lin et al., 2022). This suggests that EC enhances the resource-building potential of BJW, leading to more effective resilience development. Studies have shown that the combination of high BJW and high EC creates particularly effective resource synergies in promoting positive psychological outcomes and adaptive coping strategies (Li et al., 2022).

Drawing from the Social–Emotional Processing Model (Lemerise and Arsenio, 2000) and integrating it with COR theory, EC primarily influences how individuals process and convert their belief-based resources into resilience capabilities. Previous research has established this resource conversion link between BJW and PR, with studies suggesting that individuals with stronger BJW tend to exhibit higher levels of PR through more effective resource utilization patterns (Bartholomaeus and Strelan, 2019; Ucar et al., 2019).

Based on this resource multiplication understanding, we propose: Hypothesis 3: Empathic capacity moderates the relationship between belief in a just world and psychological resilience, such that the resource conversion process is enhanced when empathic capacity is higher.

### 1.4 The moderated mediation model

The moderated mediation model, viewed through Conservation of Resources (COR) theory, represents an integrated resource system where BJW, psychological resilience, and empathic capacity interact through sophisticated resource conversion and multiplication processes. This system explains how initial cognitive resources (BJW) are transformed into behavioral outcomes (prosocial behavior) through resource development mechanisms (PR) that vary in efficiency based on resource multipliers (EC) (Miller and Hastings, 2019).

COR theory suggests that resource dynamics operate through complex pathways where the effectiveness of resource conversion varies based on individual differences in resource configurations (Hobfoll, 2001). The strength of indirect effects through psychological resilience varies not only with EC levels but also with the specific types of resilience resources being developed and the contexts in which they are deployed. This selective enhancement explains why the indirect effect may be stronger in some situations and for certain aspects of prosocial behavior than others. When individuals possess higher levels of empathic capacity, they can more efficiently leverage their just-world beliefs to build psychological resilience, particularly in contexts requiring social–emotional resources (van den Bos and Bal, 2016).

The resource-based perspective illuminates why this moderated mediation occurs through what Hobfoll (2011) terms “resource caravans.” When individuals possess higher levels of empathic capacity, they can more effectively convert their just-world beliefs into psychological resilience resources, which in turn provides more resources for engaging in prosocial behavior. This creates what COR theory describes as resource gain spirals, where psychological resources reinforce and amplify each other’s effects (Yin and Wang, 2023).

This integrated model aligns with recent research showing that the effectiveness of psychological resources often depends on their interaction with other personal capabilities and characteristics (Li et al., 2022; Balliet et al., 2018). The model addresses calls in the literature for more complex, theoretically-grounded explanations of how personal beliefs and capabilities combine within resource systems to influence prosocial behavior.

The resource dynamics in this model operate differently across EC levels. For individuals with lower EC, the BJW-PR-PBT pathway represents what Hobfoll et al. (2018) terms a “focused resource channel,” where belief resources must be converted to resilience resources through more direct but potentially less efficient pathways. Conversely, individuals with higher EC possess what COR theory describes as “resource redundancy” (Winterich et al., 2013), allowing them to access multiple pathways for converting beliefs into resilience resources.

These differential resource patterns explain why the indirect effect of BJW on prosocial behavior through psychological resilience varies with EC levels. The moderated mediation reflects what recent research describes as “conditional resource conversion” (Zhang et al., 2024), where the efficiency of resource transformation processes depends on the availability and effectiveness of resource multipliers like empathic capacity.

Therefore, we propose: Hypothesis 4: The indirect effect of belief in a just world on prosocial behavior tendency through psychological resilience is moderated by empathic capacity, with the resource conversion process being more efficient when empathic capacity is higher.

**Figure 1:**
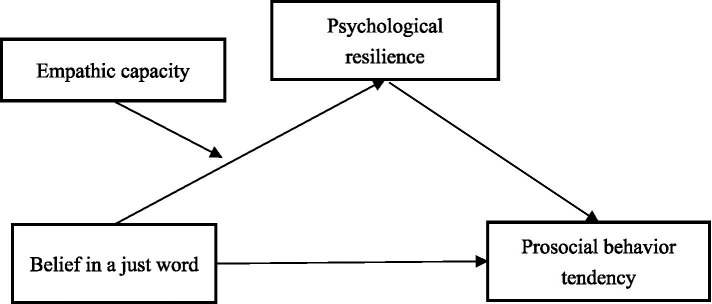
Conceptual model.

In summary, these hypotheses propose a moderated mediation model in which BJW predicts PBT, with PR acting as a mediator and EC as a moderator. It suggests that the indirect effect of BJW on PBT through PR is moderated by EC. Specifically, for individuals with lower EC, the indirect effect of BJW on PBT through PR is expected to be stronger, meaning that BJW has a greater impact on PBT through PR. Conversely, for individuals with higher EC, the indirect effect is expected to be weaker, suggesting that the impact of BJW on PBT through PR is less pronounced. This hypothesizes highlights the complex interplay between BJW, PR, EC, and PBT, and suggests that the relationship between these variables is conditional on an individual’s level of empathic capacity, as shown in Figure 1.

This study contributes to existing literature in three ways through a resource-based lens. First, it examines the psychological mechanisms through which BJW resources are converted into prosocial behavior via PR as a key resource development mechanism. Second, it investigates how individual differences in EC affect the efficiency of resource conversion processes between BJW and prosocial behavior. Third, by conducting this research in a Chinese university context, it extends our understanding of these resource dynamics beyond Western settings. These contributions help clarify the complex resource pathways leading to prosocial behavior and suggest practical implications for promoting prosocial tendencies among young adults through resource optimization strategies.

## 2 Methodology

### 2.1 Participants

After finalizing the measurement instruments, the questionnaires were entered into the Wenjuanxing (Questionnaire Star) system to generate online links for easier distribution and completion. The entire survey data collection took place between May and July 2023, with random distribution across psychology health courses in multiple majors at several universities in Sichuan Province. A total of 1,000 questionnaires were distributed, with 955 completed responses received, resulting in a 95.5% response rate. The main reasons for excluding questionnaires included excessively short response times (e.g., an average of less than 3 s per item), incomplete responses with too many missing items, or participants withdrawing mid-survey or requesting to withdraw after completion. During the survey, participants were instructed to complete the questionnaires independently and respond truthfully based on the provided guidelines. Participants had full autonomy to decide whether to discontinue or withdraw from the study and have their data deleted during or after completion. Upon finishing, participants received a small stationery gift as compensation for their participation. Prior to commencing the study, approval was obtained from the Ethics Committee of the Sichuan Psychological Association, and participants were informed about the overall process, with informed consent and voluntary participation fully respected.

The participant characteristics covered a diverse range of undergraduate majors, including education, mathematics, medicine, management, and others. Additionally, 65.6% of the participants were female, with a mean age of 19.5 ± 1.3 years. The grade level distribution was primarily from first-year to fourth-year students, with higher proportions in the first (47.3%) and second (27.7%) years. Overall, the participant sample exhibited characteristics representative of the general population of Chinese college students of the same age group, supporting the generalizability of the study’s findings to broader, similar populations to a certain extent.

### 2.2 Measure

#### 2.2.1 Prosocial tendencies measure

The Prosocial Tendencies Measure (PTM) is an instrument used to assess six types of self-reported prosocial tendencies in adolescents. The PTM was originally developed by Carlo and Randall (2002), and later translated and revised by Kou et al. (2007) to adapt it for use with Chinese adolescents based on research findings related to prosocial behavior in this population. The PTM consists of 26 items scored on a five-point scale, with 1 indicating “not at all like me” and 5 indicating “very much like me.” After revision, the PTM demonstrated good psychometric properties, with internal consistency reliabilities of 0.71 for the public subscale, 0.78 for the anonymous subscale, 0.76 for the altruistic subscale, 0.74 for the compliant subscale, 0.73 for the emotional subscale, and 0.76 for the emergency subscale.

#### 2.2.2 Belief in a Just World Scale

The Belief in a Just World Scale (BJWS) is an instrument used to measure an individual’s belief in a just world, which assesses the extent to which people believe they live in a world where people generally get what they deserve. The original scale was developed by Dalbert (2001), and a Chinese version was later revised by Su et al. in 2012. The revised scale consists of 13 items divided into two subscales: General Belief in a Just World and Personal Belief in a Just World. Responses are scored on a 6-point scale, with 6 indicating “strongly agree” and 1 indicating “strongly disagree.” The overall scale demonstrated good internal consistency, with a Cronbach’s alpha of 0.885. The subscales of Personal Belief in a Just World and General Belief in a Just World had Cronbach’s alphas of 0.794 and 0.854, respectively.

#### 2.2.3 Connor-Davidson Resilience Scale

The Connor-Davidson Resilience Scale (CD-RISC) is an instrument used to measure an individual’s resilient responses when facing adversity, trauma, tragedy, threats, or other significant life stressors. The CD-RISC was originally developed by Connor and Davidson (2003), and was later revised by Hu and Gan (2008) for use with Chinese populations. The revised CD-RISC consists of 27 items spanning five dimensions: goal-oriented, interpersonal assistance, family support, emotion control, and positive cognition. Responses are scored on a 5-point scale, with 1 indicating “not true at all” and 5 indicating “true nearly all the time.” The revised scale demonstrated good internal consistency, with a test–retest reliability of 0.83.

#### 2.2.4 Interpersonal reactivity index

The Interpersonal Reactivity Index (IRI) is a self-report questionnaire developed by Davis (1980) to measure empathy. The scale consists of 22 items across four dimensions: Fantasy Scale, Personal distress, Perspective taking, and Empathic concern. Responses are rated on a 5-point Likert scale. The perspective taking subscale assesses an individual’s tendency to adopt others’ psychological viewpoints, while the fantasy subscale measures the extent to which an individual imaginatively transposes themselves into the feelings and actions of fictitious characters. The empathic concern subscale evaluates an individual’s feelings of warmth, compassion, and concern for others, and the personal distress subscale assesses an individual’s own feelings of anxiety and discomfort when witnessing others’ negative experiences. The IRI has been widely used in research to investigate the multidimensional nature of empathy and its relationship with various psychological constructs, such as prosocial behavior (Eisenberg and Miller, 1987), emotional intelligence (Schutte et al., 2001), and moral reasoning (Skoe, 2010). The scale has demonstrated good psychometric properties, with acceptable internal consistency and test–retest reliability (Siu and Shek, 2005). In the current study, the Cronbach’s *α* coefficient for EC was 0.80, indicating good internal consistency. The internal consistency reliability coefficients for the four subscales are: Perspective Taking: *α* = 0.81; Fantasy: *α* = 0.66; Empathic Concern: *α* = 0.58; Personal Distress: *α* = 0.79. These properties make it a valuable tool for understanding the complex nature of empathy.

### 2.3 Data analysis

The data analysis strategy involved several steps using SPSS 25 and the PROCESS macro (Hayes, 2022). First, preliminary data screening was conducted to examine missing data patterns, identify outliers, and assess normality assumptions. Descriptive statistics and Pearson correlation analyses were performed to examine the relationships among all study variables (Belief in a Just World, Prosocial Behavior Tendency, Psychological Resilience, and Empathic Capacity). The hypothesized moderated mediation model was tested using Model 7 of PROCESS macro version 4.0 (Hayes, 2022). This model examines whether the indirect effect of an independent variable (BJW) on a dependent variable (PBT) through a mediator (PR) varies as a function of a moderator (EC). The analysis included: Testing the direct effect of BJW on PBT; Examining the mediating role of PR; Assessing the moderating effect of EC on the BJW-PR relationship; Testing the conditional indirect effects at different levels of EC.

Prior to main analyses, we validated the measurement structure of each construct through Confirmatory Factor Analysis (CFA) using AMOS 26.0. After confirming good model fit for all constructs (BJW (CFI = 0.959, TLI = 0.947, RMSEA = 0.075),PBT (CFI = 0.879, TLI = 0.851, RMSEA = 0.094),PR (CFI = 0.919, TLI = 0.909, RMSEA = 0.066), EC (CFI = 0.908, TLI = 0.902, RMSEA = 0.092)), we proceeded with manifest variable analysis using factor scores, which incorporates measurement model information while enabling efficient testing of our moderated mediation hypotheses through PROCESS macro.

Bootstrapping procedures with 5,000 resamples were employed to test the significance of the indirect effects and generate bias-corrected 95% confidence intervals. The index of moderated mediation was examined to determine whether the indirect effect significantly varied across levels of EC. Simple slopes analyses were conducted to probe significant interaction effects at different levels of EC (−1 SD, Mean, +1 SD).

Since this study employed questionnaire measures for all variables, common method bias was assessed using Harman’s single-factor test. This involved conducting an exploratory factor analysis (EFA) on all items. Common method bias would be indicated if a single factor emerged or if the first factor accounted for more than 50% of the variance (Podsakoff et al., 2003). The EFA results showed that the first factor accounted for 38.09% of the variance, below the 50% threshold, suggesting no severe common method bias.

## 3 Results

### 3.1 Descriptive analysis

The descriptive statistical results and partial correlations controlling for gender are shown in the Table 1 (*N* = 955). Belief in a Just World (*M* = 42.29, *SD* = 9.56) showed a weak but significant positive correlation with Prosocial Behavior Tendency (*M* = 89.15, *SD* = 15.27; *r* = 0.111, *p* < 0.001). Psychological Resilience (*M* = 78.25, *SD* = 16.82) demonstrated significant positive correlations with both Belief in a Just World (*r* = 0.352, *p* < 0.001) and Prosocial Behavior Tendency (*r* = 0.346, *p* < 0.001). Empathic Capacity (*M* = 57.58, *SD* = 12.55) was positively correlated with Belief in a Just World (*r* = 0.145, *p* < 0.001) and Prosocial Behavior Tendency (*r* = 0.242, *p* < 0.001), while showing a non-significant correlation with Psychological Resilience (*r* = 0.058, *p* > 0.05).

**Table 1:** Descriptive statistics and correlations among all variables.

Variables	*M*	SD	1	2	3	4
1. Belief in a Just World	42.29	9.56	1			
2. Prosocial Behavior Tendency	89.15	15.27	0.111***	1		
3. Psychological Resilience	78.25	16.82	0.352***	0.346***	1	
4. Empathic Capacity	57.58	12.55	0.145***	0.242***	0.058	1

*N* = 955, Controlling for gender. *p < 0.05, **p < 0.01, ***p < 0.001. M, Mean; SD, Standard Deviation.

### 3.2 Moderated meditation analysis

All moderated mediation analyses in this study were conducted while controlling for gender effects. The present study employed a moderated mediation analysis to investigate the complex relationship between Belief in a Just World (BJW) and Prosocial Behavior Tendency (PBT), with Psychological Resilience (PR) serving as a mediator and Empathic Capacity (EC) as a moderator. The analysis utilized Model 7 of the PROCESS macro (Hayes, 2022), with 5,000 bootstrap samples and a sample size of 955 participants.

**Figure 2:**
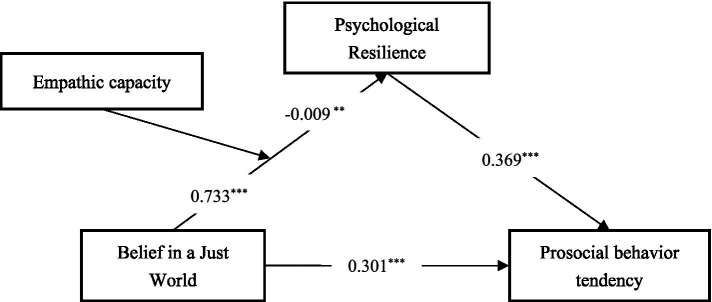
Path analysis of the moderated mediation model.

The model predicting Psychological Resilience demonstrated significant explanatory power [*F*(3, 951) = 78.287, *p* < 0.001], accounting for 24.8% of the variance in PR (*R^2^* = 0.248). Both Belief in a Just World (*β* = 0.733, *t* = 14.049, *p* < 0.001, 95% CI [0.618, 0.842]) and Empathic Capacity (β = 0.168, *t* = 4.214, *p* < 0.001, 95% CI [0.067, 0.259]) exhibited significant positive associations with PR. Notably, a significant interaction effect between BJW and EC was observed (β = −0.009, *t* = −3.061, *p* < 0.01, 95% CI [−0.020, −0.001]), indicating that the influence of BJW on PR is moderated by EC (see Table 2; Figure 2). To elucidate the nature of this moderation effect, a conditional effects analysis was conducted. The results revealed that the positive relationship between BJW and PR remained significant across all levels of EC. However, the magnitude of this relationship demonstrated a decreasing trend as EC increased, with effects of 0.83 (*p* < 0.001) at low EC, 0.72 (*p* < 0.001) at medium EC, and 0.59 (*p* < 0.001) at high EC (see Table 3).

**Table 2:** Regression model analysis.

Dependent variables	Independent variables	R	R^2^	F	β	t	Bootstrap LLCI	Bootstrap ULCI
Psychological Resilience	BJW	0.498	0.248	78.287	0.733	14.049***	0.618	0.842
	EC				0.168	4.214***	0.067	0.259
	BJW × EC				0.009	−3.061**	−0.020	−0.001
Prosocial behavior Tendency	BJW	0.506	0.256	108.882	0.301	5.763***	0.159	0.437
	PR				0.369	12.380***	0.291	0.449

**p < 0.01, ***p < 0.001. BJW, belief in a just world; EC, empathic capacity; PR, psychological resilience; LLCI and ULCI are the lowest and highest values of the confidence interval, respectively.

**Table 3:** Conditional effects of BJW on PR at different levels of EC.

EC.Level	Effect	SE	*t*	*p*	95% CI
Low (45)	0.83	0.06	13.97***	<0.001	[0.72, 0.95]
Medium (57)	0.72	0.05	13.81***	<0.001	[0.61, 0.82]
High (70)	0.59	0.07	8.77***	<0.001	[0.46, 0.72]

***p < 0.001. EC levels represent the 16th, 50th, and 84th percentiles.

The model predicting Prosocial Behavior Tendency also demonstrated significant explanatory power [*F*(2, 952) = 108.882, *p* < 0.001], accounting for 25.6% of the variance in PBT (*R^2^* = 0.256). Both BJW (β = 0.301, *t* = 5.763, *p* < 0.001, 95% CI [0.159, 0.437]) and PR (β = 0.369, *t* = 12.380, *p* < 0.001, 95% CI [0.291, 0.449]) exhibited significant positive associations with PBT (see Table 2). These results suggest that individuals with higher levels of BJW and PR tend to display greater prosocial behavior tendencies. The analysis further revealed a significant direct effect of BJW on PBT, indicating that BJW influences PBT independently of the mediating effect of PR. However, the indirect effect of BJW on PBT through PR demonstrated a more nuanced pattern, varying as a function of EC. The indirect effect was strongest at low levels of EC (Effect = 0.35, BootSE = 0.05, 95% CI [0.27, 0.46]), moderate at medium levels of EC (Effect = 0.31, BootSE = 0.04, 95% CI [0.24, 0.39]), and weakest, though still significant, at high levels of EC (Effect = 0.25, BootSE = 0.04, 95% CI [0.16, 0.33]) (see Table 4).

**Table 4:** Conditional indirect effects of BJW on PBT through PR at different levels of EC.

EC.Level	Effect	BootSE	BootLLCI	BootULCI
Low (45)	0.35	0.05	0.27	0.46
Medium (57)	0.31	0.04	0.24	0.39
High (70)	0.25	0.04	0.16	0.33

BootLLCI and BootULCI represent the lower and upper bounds of the 95% bootstrap confidence intervals.

Collectively, these findings provide empirical support for a moderated mediation model in which the relationship between Belief in a Just World and Prosocial Behavior Tendency is partially mediated by Psychological Resilience, with this mediation effect being moderated by Empathic Capacity. The results reveal a complex interplay between these constructs, highlighting the importance of considering both mediating and moderating factors in understanding the mechanisms underlying prosocial behavior tendencies. Specifically, while BJW demonstrates both direct and indirect effects on PBT, the strength of the indirect effect through PR varies inversely with EC levels. These findings contribute to a more nuanced understanding of the factors influencing prosocial behavior and underscore the need for multifaceted approaches in future research and interventions aimed at promoting prosocial tendencies.

**Figure 3:**
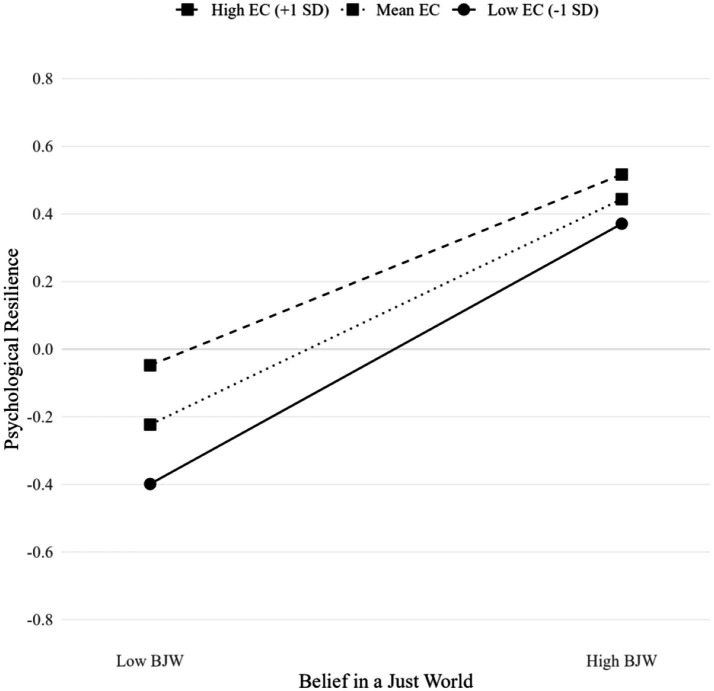
Interaction effect between belief in a just world (BJW) and empathic capacity (EC) on psychological resilience.

The simple slopes analysis, as illustrated in Figure 3, revealed a significant interaction between Belief in a Just World (BJW) and Empathic Capacity (EC) in predicting Psychological Resilience. Three distinct patterns emerged across EC levels: individuals with low EC (−1 SD) demonstrated the steepest positive slope (*b* = 0.83, SE = 0.12, *p* < 0.001, ranging from −0.40 to 0.35), indicating the strongest positive association between BJW and resilience; those with mean EC showed a moderate positive relationship (*b* = 0.72, SE = 0.09, *p* < 0.001, ranging from −0.20 to 0.45); and those with high EC (+1 SD) exhibited the flattest slope (*b* = 0.59, SE = 0.11, *p* < 0.001, ranging from −0.05 to 0.50). The divergence of slopes at low BJW levels suggests that high empathic capacity serves as a protective factor when belief in a just world is weak, while the relative convergence at high BJW levels indicates that strong belief in a just world may partially compensate for differences in empathic capacity. These findings illuminate the complex interplay between cognitive beliefs and emotional competencies in fostering psychological resilience, particularly emphasizing the compensatory effect of high empathic capacity when belief in a just world is challenged.

## 4 Discussion

In this study, a moderated mediation analysis was conducted to test four hypotheses concerning the relationships between Belief in a Just World (BJW), Prosocial Behavior Tendency (PBT), Psychological Resilience (PR), and Empathic Capacity (EC). The results provided comprehensive support for all hypotheses: BJW positively predicted PBT (H1); PR mediated the relationship between BJW and PBT (H2); EC moderated the relationship between BJW and PR, with the positive relationship being stronger for individuals with lower levels of EC (H3); and EC moderated the indirect effect of BJW on PBT through PR, with the indirect effect being stronger for individuals with lower EC levels (H4). To interpret these complex relationships, we employ Conservation of Resources (COR) theory as our overarching theoretical framework, which enables us to understand these findings through the lens of psychological resource dynamics. Within this framework, we conceptualize BJW as an initial psychological resource that facilitates the development of psychological resilience (resource development stage), which in turn enables prosocial behavior (resource deployment stage). Empathic capacity functions as a resource multiplier, selectively enhancing the efficiency of resource development processes. This resource-based perspective allows us to integrate insights from multiple theoretical traditions – including cognitive dissonance theory, self-perception theory, broaden-and-build theory, and social-cognitive theory – into a coherent explanatory framework. The following sections examine how these psychological resources interact systematically to influence prosocial behavior, moving from resource development through deployment to an integrated understanding of the complete resource system.

### 4.1 BJW as a primary resource base: resource activation and development

Within Conservation of Resources theory, belief in a just world represents a primary psychological resource that enables the acquisition and development of other resources for prosocial engagement. This foundational resource operates through three distinct resource activation and multiplication mechanisms, each contributing to the transformation of belief resources into behavioral resources.

First, through the lens of cognitive dissonance theory (Festinger, 1957), BJW activates resource deployment through psychological pressure for resource consistency. When individuals with strong just-world belief resources encounter situations that threaten their resource base (e.g., witnessing injustice), they experience what Elliot and Devine (1994) term “resource inconsistency pressure.” This pressure mobilizes existing resources toward prosocial behavior as a means of maintaining resource stability and protecting their fundamental belief resources. The resource pressure creates action-oriented states that facilitate the conversion of belief resources into behavioral resources.

Second, self-perception theory (Bem, 1972) illuminates how this resource activation creates self-reinforcing resource gain cycles. As individuals deploy their resources in prosocial actions, they interpret these behaviors as evidence supporting their just-world belief resources, creating what Kleinke and Meyer (1990) describe as “resource amplification loops.” This process explains why individuals with stronger BJW resources consistently demonstrate higher levels of prosocial engagement – their helping behaviors become integrated into their resource base, further strengthening their belief resources through what Mohiyeddini and Montada (1998) term “behavioral resource integration.”

Third, these resource dynamics manifest through specific behavioral pathways that Bègue (2014) identifies as resource investment channels. Individuals with strong BJW resources demonstrate enhanced capability in what Ucar et al. (2019) describe as “resource conversion efficiency” – the ability to transform prosocial intentions into concrete actions through optimized resource allocation. This transformation occurs through sophisticated resource deployment patterns, where just-world beliefs guide both the direction and intensity of resource investment in helping efforts. For instance, these individuals show greater persistence in prosocial activities, viewing setbacks as temporary resource challenges rather than fundamental threats to their resource base.

The direct BJW-PBT relationship also reveals distinct patterns in resource processing and response to helping opportunities. Those with stronger just-world belief resources demonstrate what Jiang et al. (2017) term “proactive resource deployment orientation” – they actively seek opportunities to invest their resources in helping others rather than merely responding to explicit resource demands. This proactive orientation stems from their fundamental belief that prosocial resource investments contribute to maintaining a just world, creating what COR theory describes as a more stable and predictable resource environment.

### 4.2 The resource transformation process: psychological resilience as a resource converter

Within COR theory, psychological resilience functions as a critical resource conversion mechanism, transforming initial belief resources (BJW) into sustainable prosocial behavioral resources. Broaden-and-build theory (Fredrickson, 2001) illuminates how BJW resources generate positive emotional experiences that build psychological resilience resources. When individuals encounter challenges in resource deployment during helping situations, their just-world belief resources activate what Bartholomaeus and Strelan (2019) identify as “resource-building appraisals.” These appraisals manifest in three distinct resource enhancement processes: accelerated recovery of depleted resources after helping activities, improved resource conservation during challenging prosocial encounters, and increased capacity for sustained resource deployment despite temporary depletion. Through repeated activation, these processes create what Fredrickson (2013) terms “stable resource reservoirs,” enabling individuals to maintain prosocial resource investment even under conditions of resource stress.

Social-cognitive theory (Bandura, 1991) further explicates the specific resource conversion mechanisms through which psychological resilience facilitates prosocial behavior. Cheng et al. (2020) identify three sequential and interactive resource management processes. First, strategic resource allocation involves optimizing the distribution of available resources across helping goals based on resource capacity assessment. Second, resource mobilization encompasses selecting efficient resource deployment strategies and adapting them to specific situational resource demands. Third, resource maintenance focuses on sustaining helping behavior under pressure while implementing what Wu et al. (2018) describe as “resource conservation strategies.” These processes explain why individuals with higher psychological resilience resources demonstrate more sustained prosocial engagement, particularly in situations requiring long-term resource commitment or facing significant resource barriers.

The resource conversion process also operates through sophisticated resource reappraisal mechanisms (Gross and John, 2003), where resilient individuals transform potentially resource-depleting helping situations into opportunities for resource growth and expansion. This transformation process, as documented by Li et al. (2022), involves three distinct resource enhancement components: cognitive restructuring of resource challenges, emotional resource regulation during helping encounters, and resource recovery optimization after prosocial engagement. These components work together to create what COR theory terms “resource gain spirals,” reducing resource exhaustion and enhancing resource recovery from helping-related stress, thereby creating more sustainable patterns of prosocial resource deployment that can be maintained over extended periods.

### 4.3 The resource regulation function: empathic capacity as resource multiplier and moderator

Within COR theory, empathic capacity functions as a sophisticated resource multiplier and regulatory mechanism, determining the efficiency of resource conversion between just-world beliefs and psychological resilience. Through moral identity theory (Aquino and Reed II., 2002), individuals with higher empathic capacity possess expanded resource portfolios that include advanced emotional recognition resources, sophisticated perspective-taking capabilities, and enhanced social sensitivity mechanisms. Hardy et al. (2014) demonstrate how these additional resources create multiple independent pathways for developing psychological resilience, reducing their reliance on just-world beliefs alone. High-EC individuals can simultaneously process others’ emotional states, maintain emotional boundaries, and regulate their helping responses – capabilities that Eisenberg et al. (2010) show contribute directly to psychological resilience independent of just-world beliefs.

The differential strength of the BJW-PR relationship across EC levels emerges from distinct patterns in resource acquisition and utilization efficiency. Zhang et al. (2020) reveal that high-EC individuals demonstrate superior resource acquisition capabilities through three mechanisms: rapid emotional information processing, efficient emotional resource conservation, and accelerated resource recovery after helping episodes. These capabilities allow them to build psychological resilience through multiple complementary channels, including direct emotional processing, social connection, and meaning-making processes. Mohiyeddini and Montada (1998) show that these individuals can maintain psychological stability even when their just-world beliefs are challenged, as their emotional resources provide alternative support mechanisms.

Conversely, individuals with lower empathic capacity demonstrate what Staub (2015) terms “resource-restricted resilience.” Without sophisticated emotional processing resources, they rely heavily on cognitive belief systems for psychological stability. This dependency manifests in three specific ways: slower recovery from emotional exhaustion during helping situations (Ito and Brotheridge, 2003), greater vulnerability to emotional contagion when witnessing others’ distress (Balconi and Canavesio, 2013), and reduced capacity for simultaneous helping demands (Ruci et al., 2018). These limitations make just-world beliefs critical for their psychological resilience, explaining the stronger mediation effect in this group.

The resource substitution patterns further illuminate the moderating effect. High-EC individuals can substitute emotional resources for belief-based resources when facing challenges to their just-world beliefs, maintaining psychological resilience through what Tajfel and Turner (1979) identify as flexible resource deployment. In contrast, low-EC individuals show limited resource substitution capabilities, leading to what Hoyt and Price (2015) term “resource rigidity” – an inability to compensate for belief system challenges through alternative resource channels. This rigidity makes the relationship between their just-world beliefs and psychological resilience more pronounced but potentially more vulnerable to disruption.

### 4.4 The integrated resource system: understanding resource network dynamics

Through COR theory, the moderated mediation effect represents a dynamic resource ecosystem where BJW, psychological resilience, and empathic capacity interact through sophisticated resource exchange networks. Hayes’ (2018) framework helps us understand how these resources combine to create what we term “differential resource cascades” – distinct patterns of prosocial resource deployment that vary systematically across individual resource portfolios and situational contexts. Lin et al. (2022) demonstrate how these resource cascades explain variations in prosocial consistency through multiple interactive processes operating simultaneously at cognitive, emotional, and behavioral resource levels.

For individuals with lower empathic capacity, the BJW-PR-PBT pathway represents what Eisenberg et al. (2010) identify as a “concentrated resource channel.” This resource concentration manifests through intensified reliance on belief-based resource motivation, heightened resource vulnerability during belief system challenges, and variable resource deployment efficiency across contexts. These characteristics explain why these individuals demonstrate what COR theory terms “resource deployment fluctuation” – their prosocial behavior shows greater variation across situations due to heavy dependence on single-channel resource pathways. The concentrated nature of their resource system makes them particularly susceptible to resource depletion when facing sustained helping demands or multiple simultaneous challenges.

In contrast, high-EC individuals demonstrate what Winterich et al. (2013) describe as “resource redundancy” – multiple parallel resource pathways that create stable foundations for prosocial behavior. This resource redundancy operates through parallel processing of resource deployment opportunities, simultaneous activation of multiple resource motivation channels, and flexible resource adaptation across helping contexts. These mechanisms explain the maintenance of consistent helping patterns through what COR theory identifies as “resource buffering” – the ability to sustain prosocial engagement through multiple resource reserves. High-EC individuals can maintain stable prosocial behavior even when specific resource channels are temporarily depleted or challenged.

The integrated resource system reveals a complex interplay between primary and secondary resource networks. BJW functions as a foundational resource base, providing the initial resources necessary for prosocial engagement. Psychological resilience serves as a sophisticated resource conversion mechanism, transforming these initial resources into sustainable behavioral patterns. Empathic capacity operates as both resource multiplier and regulator, determining the efficiency and flexibility of resource utilization across different contexts. This integrated perspective demonstrates how multiple resource pathways support prosocial behavior through complementary mechanisms, creating what we term “resource stability gradients” – varying levels of sustainable prosocial engagement based on individual resource portfolios and environmental demands.

### 4.5 Implications

Our moderated mediation model generates significant theoretical and practical implications for understanding and promoting prosocial behavior development. The model advances theoretical understanding by revealing the complex interplay between cognitive, emotional, and behavioral factors in shaping prosocial tendencies (Aquino et al., 2009). The finding that EC moderates the indirect effect of BJW on PBT through PR demonstrates that prosocial behavior emerges through differentiated pathways depending on individual characteristics (Decety and Jackson, 2004). This multi-pathway model challenges single-factor explanations of prosocial behavior and suggests a more nuanced understanding of how personal resources interact to produce helping behavior. The varying influence of BJW across different EC levels, being more pronounced in individuals with lower EC (Bègue, 2014; Lerner, 1980), highlights the compensatory nature of psychological resources in prosocial development. This advances our understanding of the psychological mechanisms driving prosocial behavior (Batson, 2011) by revealing how different resource configurations can lead to similar prosocial outcomes through distinct developmental trajectories.

The model illuminates the dynamic nature of prosocial resource development. When BJW operates through psychological resilience, it creates resource amplification cycles, where initial prosocial actions strengthen both belief systems and resilience capacity, leading to more sustained prosocial engagement. This cyclical process helps explain the stability of prosocial tendencies over time and across situations. The findings suggest that prosocial behavior emerges not merely from isolated psychological factors but from sophisticated interactions between beliefs, emotional capacities, and behavioral tendencies, creating self-reinforcing patterns of helping behavior.

Our findings advocate for differentiated approaches to fostering prosocial behavior (Decety and Cowell, 2014). For individuals with lower EC, interventions should focus on strengthening BJW and PR through cognitive-behavioral techniques that enhance personal control and resilience (Furnham, 2003; Lerner, 1980). These techniques might include structured reflection exercises on successful helping experiences, progressive goal-setting in prosocial activities, and resilience-building through graduated helping challenges. Conversely, for those with higher EC, interventions should emphasize empathy and perspective-taking skills through active listening and role-playing exercises (Batson et al., 2015), including advanced emotional regulation training and engagement with diverse helping contexts.

Organizations and educational institutions can implement these insights through comprehensive development programs that assess individual resource profiles and create tailored interventions. These programs should incorporate progressive challenges that match individual resource levels while monitoring intervention effectiveness. The model suggests developing age-appropriate prosocial development curricula that integrate belief-strengthening and empathy-building activities, creating sustainable pathways for prosocial development. This tailored approach promises more effective outcomes across different individual profiles (Penner et al., 2005; Eisenberg et al., 2010) by matching interventions to individual resource configurations and enabling long-term behavior change through resource optimization.

### 4.6 Limitations and future research

Several limitations warrant consideration in interpreting our findings. First, our reliance on self-report measures may introduce social desirability bias (Aquino et al., 2009). Second, the cross-sectional design limits causal inferences about the relationships between variables (Maxwell and Cole, 2007). Third, our sample of Chinese college students raises questions about generalizability to other populations (Henrich et al., 2010). Finally, while comprehensive, our model necessarily excludes some potential influences on prosocial behavior, such as personality traits and situational factors (Penner et al., 2005).

Future research should address these limitations through multiple approaches. Longitudinal studies are needed to track the development and interaction of these variables over time, providing stronger evidence for causal relationships (Ployhart and Vandenberg, 2010). The integration of neurophysiological measures could provide objective indicators of emotional and cognitive processes, complementing self-report data (Lieberman, 2007). Additionally, cross-cultural investigations would help determine whether the identified moderated mediation effects are universal or culturally specific (Markus and Kitayama, 2014). Research should also explore additional moderating factors, such as moral reasoning and cultural values (Funder, 2009), while intervention studies could test practical applications for enhancing prosocial behavior across different EC levels (Walton, 2014). These directions would not only address current limitations but also advance our understanding of prosocial behavior development and promotion across diverse contexts.

## 5 Conclusion

In conclusion, our study reveals how Belief in a Just World influences Prosocial Behavior Tendency through Psychological Resilience, with this relationship being moderated by Empathic Capacity. Our findings demonstrate that this indirect effect is stronger for individuals with lower EC and weaker for those with higher EC. These results advance our theoretical understanding of prosocial behavior by identifying distinct pathways through which beliefs and emotional capacities interact to shape prosocial tendencies. Practically, these findings suggest that interventions to promote prosocial behavior should be tailored based on individual differences in empathic capacity, with different approaches needed for high versus low EC individuals. This research provides a foundation for developing more effective, personalized approaches to fostering prosocial behavior across diverse populations.

## Data Availability

The raw data supporting the conclusions of this article will be made available by the authors, without undue reservation.

## References

[ref1] An Y. Lu A. Chen W. Xue S. Ke X. Li J.. (2024). Transcending belief: exploring the impact of belief in a just world on self-regulated learning in Chinese adolescents using latent transitions analysis. Psychol. Res. Behav. Manag. 17, 3691–3708. doi: 10.2147/PRBM.S473451, PMID: 39469222 PMC11514654

[ref2] Aquino K. Freeman D. Reed A. II Lim V. K. Felps W. (2009). Testing a social-cognitive model of moral behavior: the interactive influence of situations and moral identity centrality. J. Pers. Soc. Psychol. 97, 123–141. doi: 10.1037/a0015406, PMID: 19586244

[ref3] Aquino K. Reed A. II. (2002). The self-importance of moral identity. J. Pers. Soc. Psychol. 83, 1423–1440. doi: 10.1037/0022-3514.83.6.1423, PMID: 12500822

[ref4] Balconi M. Canavesio Y. (2013). Emotional contagion and trait empathy in prosocial behavior in young people: the contribution of autonomic (facial feedback) and balanced emotional empathy scale (BEES) measures. J. Clin. Exp. Neuropsychol. 35, 41–48. doi: 10.1080/13803395.2012.742492, PMID: 23157445

[ref5] Balliet D. Parks C. Joireman J. (2018). Social value orientation and cooperation in social dilemmas: a meta-analysis. Group Process. Intergroup Relat. 12, 533–547. doi: 10.1177/1368430209105040, PMID: 39935846

[ref6] Bandura A. (1991). Social cognitive theory of self-regulation. Organ. Behav. Hum. Decis. Process. 50, 248–287. doi: 10.1016/0749-5978(91)90022-L

[ref8] Bartholomaeus J. Strelan P. (2019). The adaptive, approach-oriented correlates of belief in a just world for the self: a review of the research. Personal. Individ. Differ. 151:109485. doi: 10.1016/j.paid.2019.06.028

[ref9] Batson C. D. (2011). Altruism in humans. Oxford: Oxford University Press.

[ref10] Batson C. D. Lishner D. A. Stocks E. L. (2015). “The empathy-altruism hypothesis” in The Oxford handbook of prosocial behavior (Oxford: Oxford University Press), 259–281.

[ref11] Bègue L. (2014). Do just-world believers practice private charity? J. Appl. Soc. Psychol. 44, 71–76. doi: 10.1111/jasp.12201

[ref12] Bègue L. Charmoillaux M. Cochet J. Cury C. De Suremain F. (2008). Altruistic behavior and the bidimensional just world belief. Am. J. Psychol. 121, 47–56. doi: 10.2307/20445443, PMID: 18437801

[ref13] Bem D. J. (1972). “Self-perception theory” in Advances in experimental social psychology. ed. Berkowitz L., vol. 6 (Cambridge, MA: Academic Press), 1–62.

[ref14] Biglan A. Johansson M. Van Ryzin M. Embry D. (2020). Scaling up and scaling out: consilience and the evolution of more nurturing societies. Clin. Psychol. Rev. 81:101893. doi: 10.1016/j.cpr.2020.101893, PMID: 32858377 PMC7403031

[ref9002] Carlo G. Randall B. A. (2002). The development of a measure of prosocial behaviors for late adolescents. J. Youth Adolesc. 31, 31–44. doi: 10.1023/A:1014033032440

[ref15] Cheng Y. Nudelman G. Otto K. Ma J. (2020). Belief in a just world and employee voice behavior: the mediating roles of perceived efficacy and risk. J. Psychol. 154, 129–143. doi: 10.1080/00223980.2019.1670126, PMID: 31644371

[ref16] Chobthamkit P. Sutton R. M. English A. S. Wongvorachan T. Datu J. A. D. Chung K. L.. (2024). Belief in a just world for the self and others, karma, system justification and well-being during COVID-19 pandemic: evidence from 15 Asian nations. Asian J. Soc. Psychol. 28:e12667. doi: 10.1111/ajsp.12667, PMID: 39967264

[ref9003] Connor K. M. Davidson J. R. (2003). Development of a new resilience scale: The Connor‐Davidson resilience scale (CD‐RISC). Depression and Anxiety 18, 76–82. doi: 10.1002/da.10113 12964174

[ref18] Curry O. S. Rowland L. A. Van Lissa C. J. Zlotowitz S. McAlaney J. Whitehouse H. (2018). Happy to help? A systematic review and meta-analysis of the effects of performing acts of kindness on the well-being of the actor. J. Exp. Soc. Psychol. 76, 320–329. doi: 10.1016/j.jesp.2018.02.014

[ref19] Dalbert C. (2001). The justice motive as a personal resource: dealing with challenges and critical life events. New York, NY: Kluwer Academic/Plenum Publishers.

[ref20] Datu J. A. D. Restubog S. L. D. (2020). The emotional pay-off of staying gritty: linking grit with social-emotional learning and emotional well-being. British J Guidance Counsel 48, 697–708. doi: 10.1080/03069885.2020.1758922

[ref21] Davis M. H. (1980). “A multidimensional approach to individual differences in empathy” in JSAS Catalog of Selected Documents in Psychology, vol. 10, 85.

[ref22] Davis M. H. (1983). Measuring individual differences in empathy: evidence for a multidimensional approach. J. Pers. Soc. Psychol. 44, 113–126. doi: 10.1037/0022-3514.44.1.113

[ref23] Decety J. Bartal I. B. A. Uzefovsky F. Knafo-Noam A. (2016). Empathy as a driver of prosocial behaviour: highly conserved neurobehavioural mechanisms across species. Philos. Trans. Royal Society B 371:20150077. doi: 10.1098/rstb.2015.0077, PMID: 26644596 PMC4685523

[ref24] Decety J. Cowell J. M. (2014). The complex relation between morality and empathy. Trends Cogn. Sci. 18, 337–339. doi: 10.1016/j.tics.2014.04.008 24972506

[ref25] Decety J. Jackson P. L. (2004). The functional architecture of human empathy. Behav. Cogn. Neurosci. Rev. 3, 71–100. doi: 10.1177/1534582304267187, PMID: 15537986

[ref27] Eisenberg N. Eggum N. D. Di Giunta L. (2010). Empathy-related responding: associations with prosocial behavior, aggression, and intergroup relations. Soc. Issues Policy Rev. 4, 143–180. doi: 10.1111/j.1751-2409.2010.01020.x, PMID: 21221410 PMC3017348

[ref28] Eisenberg N. Miller P. A. (1987). The relation of empathy to prosocial and related behaviors. Psychol. Bull. 101, 91–119. doi: 10.1037/0033-2909.101.1.91 3562705

[ref29] Elliot A. J. Devine P. G. (1994). On the motivational nature of cognitive dissonance: dissonance as psychological discomfort. J. Pers. Soc. Psychol. 67, 382–394. doi: 10.1037/0022-3514.67.3.382

[ref30] Festinger L. (1957). A theory of cognitive dissonance. Redwood City, CA: Stanford University Press.

[ref31] Fletcher D. Sarkar M. (2013). Psychological resilience: a review and critique of definitions, concepts, and theory. Eur. Psychol. 18, 12–23. doi: 10.1027/1016-9040/a000124

[ref32] Fredrickson B. L. (2001). The role of positive emotions in positive psychology: the broaden-and-build theory of positive emotions. Am. Psychol. 56, 218–226. doi: 10.1037/0003-066X.56.3.218, PMID: 11315248 PMC3122271

[ref33] Fredrickson B. L. (2013). “Positive emotions broaden and build” in Advances in experimental social psychology. eds. Devine P. Plant A., vol. 47 (Cambridge, MA: Academic Press), 1–53.

[ref34] Funder D. C. (2009). Persons, behaviors and situations: An agenda for personality psychology in the postwar era. J. Res. Pers. 43, 120–126. doi: 10.1016/j.jrp.2008.12.041

[ref35] Furnham A. (2003). Belief in a just world: research progress over the past decade. Personal. Individ. Differ. 34, 795–817. doi: 10.1016/S0191-8869(02)00072-7

[ref9001] Gross J. J. John O. P. (2003). Individual differences in two emotion regulation processes: implications for affect, relationships, and well-being. J. Pers. Soc. Psychol. 85:348. doi: 10.1037/0022-3514.85.2.348 12916575

[ref36] Guo Y. Chen X. Ma J. Li Y. Hommey C. (2022). How belief in a just world leads to prosocial behaviours: the role of communal orientation. Personal. Individ. Differ. 195:111642. doi: 10.1016/j.paid.2022.111642

[ref37] Hafer C. L. Sutton R. (2016). “Belief in a just world” in Handbook of social justice theory and research. eds. Sabbagh C. Schmitt M. (Berlin: Springer), 145–160.

[ref38] Hardy S. A. Walker L. J. Olsen J. A. Woodbury R. D. Hickman J. R. (2014). Moral identity as moral ideal self: links to adolescent outcomes. Dev. Psychol. 50, 45–57. doi: 10.1037/a0033598, PMID: 23895167

[ref39] Hayes A. F. (2018). Partial, conditional, and moderated moderated mediation: quantification, inference, and interpretation. Commun. Monogr. 85, 4–40. doi: 10.1080/03637751.2017.1352100

[ref40] Hayes A. F. (2022). Introduction to mediation, moderation, and conditional process analysis: A regression-based approach. 3rd Edn. New York, NY: The Guilford Press.

[ref41] Henrich J. Heine S. J. Norenzayan A. (2010). The weirdest people in the world? Behav. Brain Sci. 33, 61–83. doi: 10.1017/S0140525X0999152X, PMID: 20550733

[ref9004] Hobfoll S. E. (1989). Conservation of resources: a new attempt at conceptualizing stress. Am. Psychol. 44:513. 2648906 10.1037//0003-066x.44.3.513

[ref9005] Hobfoll S. E. (2001). The influence of culture, community, and the nested‐self in the stress process: Advancing conservation of resources theory. Appl. Psychol. 50, 337–421. doi: 10.1111/1464-0597.00062

[ref9006] Hobfoll S. E. (2002). Social and psychological resources and adaptation. Rev. Gen. Psychol. 6, 307–324. doi: 10.1037/1089-2680.6.4.307

[ref42] Hobfoll S. E. (2011). “Conservation of resources theory: its implication for stress, health, and resilience” in The Oxford handbook of stress, health, and coping, vol. 127 (Oxford: Oxford University Press), 147.

[ref43] Hobfoll S. E. Halbesleben J. Neveu J. P. Westman M. (2018). Conservation of resources in the organizational context: the reality of resources and their consequences. Annu. Rev. Organ. Psych. Organ. Behav. 5, 103–128. doi: 10.1146/annurev-orgpsych-032117-104640

[ref44] Hoyt C. L. Price T. L. (2015). Ethical decision making and leadership: merging social role and self-construal perspectives. J. Bus. Ethics 126, 531–539. doi: 10.1007/s10551-013-1974-x

[ref45] Hui B. P. H. Ng J. C. K. Berzaghi E. Cunningham-Amos L. A. Kogan A. (2020). Rewards of kindness? A meta-analysis of the link between prosociality and well-being. Psychol. Bull. 146, 1084–1116. doi: 10.1037/bul0000298, PMID: 32881540

[ref9007] Hu Y. Q. Gan Y. Q. (2008). Development and psychometric validity of the resilience scale for Chinese adolescents. Acta Psychologica Sinica. doi: 10.3724/SP.J.1041.2008.00902

[ref46] Ito J. K. Brotheridge C. M. (2003). Resources, coping strategies, and emotional exhaustion: a conservation of resources perspective. J. Vocat. Behav. 63, 490–509. doi: 10.1016/S0001-8791(02)00033-7

[ref47] Jiang H. Chen G. Wang T. (2017). Relationship between belief in a just world and internet altruistic behavior in a sample of Chinese undergraduates: multiple mediating roles of gratitude and self-esteem. Personal. Individ. Differ. 104, 493–498. doi: 10.1016/j.paid.2016.09.005

[ref48] Kındap-Tepe Y. Aktaş V. (2021). The mediating role of needs satisfaction for prosocial behavior and autonomy support. Curr. Psychol. 40, 5212–5224. doi: 10.1007/s12144-019-00466-9

[ref49] Kleinke C. L. Meyer C. (1990). Evaluation of rape victim by men and women with high and low belief in a just world. Psychol. Women Q. 14, 343–353. doi: 10.1111/j.1471-6402.1990.tb00024.x

[ref9008] Kou Y. Hong H. F. Tan C. Li L. (2007). Revision of prosocial tendency Scale for adolescents. Psychol. Dev. Educ. 23, 112–117.

[ref9009] Lemerise E. A. Arsenio W. F. (2000). An integrated model of emotion processes and cognition in social information processing. Child Dev. 71, 107–118. doi: 10.1111/1467-8624.00124 10836564

[ref50] Lerner M. J. (1980). The belief in a just world: A fundamental delusion. New York, NY: Plenum Press.

[ref51] Li X. Guan L. Chang H. Zhang B. (2014). Core self-evaluation and burnout among nurses: the mediating role of coping styles. PLoS One 9:e115799. doi: 10.1371/journal.pone.0115799, PMID: 25541990 PMC4277418

[ref52] Li L. Liu H. Wang G. Chen Y. Huang L. (2022). The relationship between ego depletion and prosocial behavior of college students during the COVID-19 pandemic: the role of social self-efficacy and personal belief in a just world. Front. Psychol. 13:801006. doi: 10.3389/fpsyg.2022.801006, PMID: 35548506 PMC9083063

[ref54] Lieberman M. D. (2007). Social cognitive neuroscience: a review of core processes. Annu. Rev. Psychol. 58, 259–289. doi: 10.1146/annurev.psych.58.110405.085654, PMID: 17002553

[ref55] Lim D. DeSteno D. (2016). Suffering and compassion: the links among adverse life experiences, empathy, compassion, and prosocial behavior. Emotion 16, 175–182. doi: 10.1037/emo0000144, PMID: 26751630

[ref56] Lin S. H. J. Poulton E. C. Tu M. H. Xu M. (2022). The consequences of empathic concern for the actors themselves: understanding empathic concern through conservation of resources and work-home resources perspectives. J. Appl. Psychol. 107, 1843–1863. doi: 10.1037/apl0000984, PMID: 34941290

[ref58] Markus H. R. Kitayama S. (2014). “Culture and the self: implications for cognition, emotion, and motivation” in College student development and academic life (London: Routledge), 264–293.

[ref59] Maxwell S. E. Cole D. A. (2007). Bias in cross-sectional analyses of longitudinal mediation. Psychol. Methods 12, 23–44. doi: 10.1037/1082-989x.12.1.23, PMID: 17402810

[ref60] Miller J. G. Hastings P. D. (2019). “Parenting, neurobiology, and prosocial development” in The Oxford handbook of parenting and moral development. eds. Laible D. J. Carlo G. Padilla-Walker L. M. (Oxford: Oxford University Press), 129–144.

[ref61] Mohiyeddini C. Montada L. (1998). “BJW and self-efficacy in coping with observed victimization: results from a study about unemployment” in Responses to victimizations and belief in a just world. eds. Montada L. Lerner M. J. (New York, NY: Plenum Press), 41–54.

[ref62] Penner L. A. Dovidio J. F. Piliavin J. A. Schroeder D. A. (2005). Prosocial behavior: multilevel perspectives. Annu. Rev. Psychol. 56, 365–392. doi: 10.1146/annurev.psych.56.091103.070141, PMID: 15709940

[ref63] Ployhart R. E. Vandenberg R. J. (2010). Longitudinal research: the theory, design, and analysis of change. J. Manag. 36, 94–120. doi: 10.1177/0149206309352110

[ref64] Podsakoff P. M. MacKenzie S. B. Lee J. Y. Podsakoff N. P. (2003). Common method biases in behavioral research: a critical review of the literature and recommended remedies. J. Appl. Psychol. 88, 879–903. doi: 10.1037/0021-9010.88.5.879, PMID: 14516251

[ref66] Reinhardt N. Reinhard M.-A. Schindler S. (2023). Is peoples’ belief in a just world associated with (dis)honesty in romantic relationships? J. Res. Pers. 105:104396. doi: 10.1016/j.jrp.2023.104396

[ref67] Ruci L. van Allen Z. M. Zelenski J. M. (2018). Pro-social personality traits, helping behavior, and ego-depletion: is helping really easier for the dispositionally pro-social? Personal. Individ. Differ. 120, 32–39. doi: 10.1016/j.paid.2017.08.013

[ref68] Rullo M. Lalot F. Heering M. S. (2022). Moral identity, moral self-efficacy, and moral elevation: a sequential mediation model predicting moral intentions and behaviour. J. Posit. Psychol. 17, 545–560. doi: 10.1080/17439760.2021.1871942

[ref69] Schutte N. S. Malouff J. M. Bobik C. Coston T. D. Greeson C. Jedlicka C.. (2001). Emotional intelligence and interpersonal relations. J. Soc. Psychol. 141, 523–536. doi: 10.1080/00224540109600569 11577850

[ref72] Serrano-Montilla C. Alonso-Ferres M. Navarro-Carrillo G. Lozano L. M. Valor-Segura I. (2021). Assessment of the effects of health and financial threat on prosocial and antisocial responses during the COVID-19 pandemic: the mediating role of empathic concern. Personal. Individ. Differ. 178:110855. doi: 10.1016/j.paid.2021.110855, PMID: 36540784 PMC9755893

[ref73] Siu A. M. H. Shek D. T. L. (2005). Validation of the interpersonal reactivity index in a Chinese context. Res. Soc. Work. Pract. 15, 118–126. doi: 10.1177/1049731504270384

[ref74] Skoe E. E. A. (2010). The relationship between empathy-related constructs and care-based moral development in young adulthood. J. Moral Educ. 39, 191–211. doi: 10.1080/03057241003754930

[ref75] Staub E. (2015). The roots of goodness and resistance to evil: Inclusive caring, moral courage, altruism born of suffering, active bystandership, and heroism. Oxford: Oxford University Press. 16335738

[ref76] Tajfel H. Turner J. C. (1979). “An integrative theory of intergroup conflict” in The social psychology of intergroup relations. eds. Austin W. G. Worchel S. (Pacific Grove, CA: Brooks/Cole), 33–47.

[ref77] Tian Y. Song Y. Zhou Z. Huang C. Zhao Y. (2019). A multilevel analysis of the developmental trajectory of belief in a just world in adolescence and its predictors. Psychol. Dev. Educ. 35, 145–154. doi: 10.16187/j.cnki.issn1001-4918.2019.02.03

[ref78] Ucar G. K. Hasta D. Malatyali M. K. (2019). The mediating role of perceived control and hopelessness in the relation between personal belief in a just world and life satisfaction. Personal. Individ. Differ. 143, 68–73. doi: 10.1016/j.paid.2019.02.021

[ref80] van den Bos K. Bal M. (2016). “Social-cognitive and motivational processes underlying the justice motive” in Handbook of social justice theory and research. eds. Sabbagh C. Schmitt M. (New York, NY: Springer).

[ref81] Van der Graaff J. Carlo G. Crocetti E. Koot H. M. Branje S. (2018). Prosocial behavior in adolescence: gender differences in development and links with empathy. J. Youth Adolesc. 47, 1086–1099. doi: 10.1007/s10964-017-0786-1, PMID: 29185207 PMC5878203

[ref82] Walton G. M. (2014). The new science of wise psychological interventions. Curr. Dir. Psychol. Sci. 23, 73–82. doi: 10.1177/0963721413512856

[ref83] Winterich K. P. Aquino K. Mittal V. Swartz R. (2013). When moral identity symbolization motivates prosocial behavior: the role of recognition and moral identity internalization. J. Appl. Psychol. 98, 759–770. doi: 10.1037/a0033177, PMID: 23751218

[ref84] Wu T. Deng Z. Feng Z. Gaskin D. J. Zhang D. Wang R. (2018). The effect of doctor-consumer interaction on social media on consumers’ health behaviors: cross-sectional study. J. Med. Internet Res. 20:e9003. doi: 10.2196/jmir.9003, PMID: 29490892 PMC5852273

[ref85] Yin Y. Wang Y. (2023). Is empathy associated with more prosocial behaviour? A meta-analysis. Asian J. Soc. Psychol. 26, 3–22. doi: 10.1111/ajsp.12537

[ref87] Zhang H. Zhu H. Jia X. Ma C. (2024). The influence of subjective social status on adolescents’ prosocial risk-taking behavior: a moderated chain mediation model. Psychol. Dev. Educ. 40, 488–498. doi: 10.16187/j.cnki.issn1001-4918.2024.04.04

[ref86] Zhang J. W. Chen S. Tomova Shakur T. K. (2020). From me to you: self-compassion predicts acceptance of own and others’ imperfections. Personal. Soc. Psychol. Bull. 46, 228–242. doi: 10.1177/0146167219853846, PMID: 31185807

